# Automatic stridor detection using small training set via patch-wise few-shot learning for diagnosis of multiple system atrophy

**DOI:** 10.1038/s41598-023-37620-0

**Published:** 2023-07-05

**Authors:** Jong Hyeon Ahn, Ju Hwan Lee, Chae Yeon Lim, Eun Yeon Joo, Jinyoung Youn, Myung Jin Chung, Jin Whan Cho, Kyungsu Kim

**Affiliations:** 1 Department of Neurology, Samsung Medical Center, Sungkyunkwan University School of Medicine, Seoul, Republic of Korea; 2 Neuroscience Center, Samsung Medical Center, Seoul, Republic of Korea; 3 Department of Health Sciences and Technology, SAIHST, Sungkyunkwan University, Seoul, Republic of Korea; 4 Department of Medical Device Management and Research, SAIHST, Sungkyunkwan University, Seoul, Republic of Korea; 5 Medical AI Research Center, Research Institute for Future Medicine, Samsung Medical Center, Seoul, Republic of Korea; 6 Department of Data Convergence and Future Medicine, Sungkyunkwan University School of Medicine, Seoul, Republic of Korea

**Keywords:** Diagnosis, Neurology

## Abstract

Stridor is a rare but important non-motor symptom that can support the diagnosis and prediction of worse prognosis in multiple system atrophy. Recording sounds generated during sleep by video-polysomnography is recommended for detecting stridor, but the analysis is labor intensive and time consuming. A method for automatic stridor detection should be developed using technologies such as artificial intelligence (AI) or machine learning. However, the rarity of stridor hinders the collection of sufficient data from diverse patients. Therefore, an AI method with high diagnostic performance should be devised to address this limitation. We propose an AI method for detecting patients with stridor by combining audio splitting and reintegration with few-shot learning for diagnosis. We used video-polysomnography data from patients with stridor (19 patients with multiple system atrophy) and without stridor (28 patients with parkinsonism and 18 patients with sleep disorders). To the best of our knowledge, this is the first study to propose a method for stridor detection and attempt the validation of few-shot learning to process medical audio signals. Even with a small training set, a substantial improvement was achieved for stridor detection, confirming the clinical utility of our method compared with similar developments. The proposed method achieved a detection accuracy above 96% using data from only eight patients with stridor for training. Performance improvements of 4%–13% were achieved compared with a state-of-the-art AI baseline. Moreover, our method determined whether a patient had stridor and performed real-time localization of the corresponding audio patches, thus providing physicians with support for interpreting and efficiently employing the results of this method.

## Introduction

**Figure 1:**
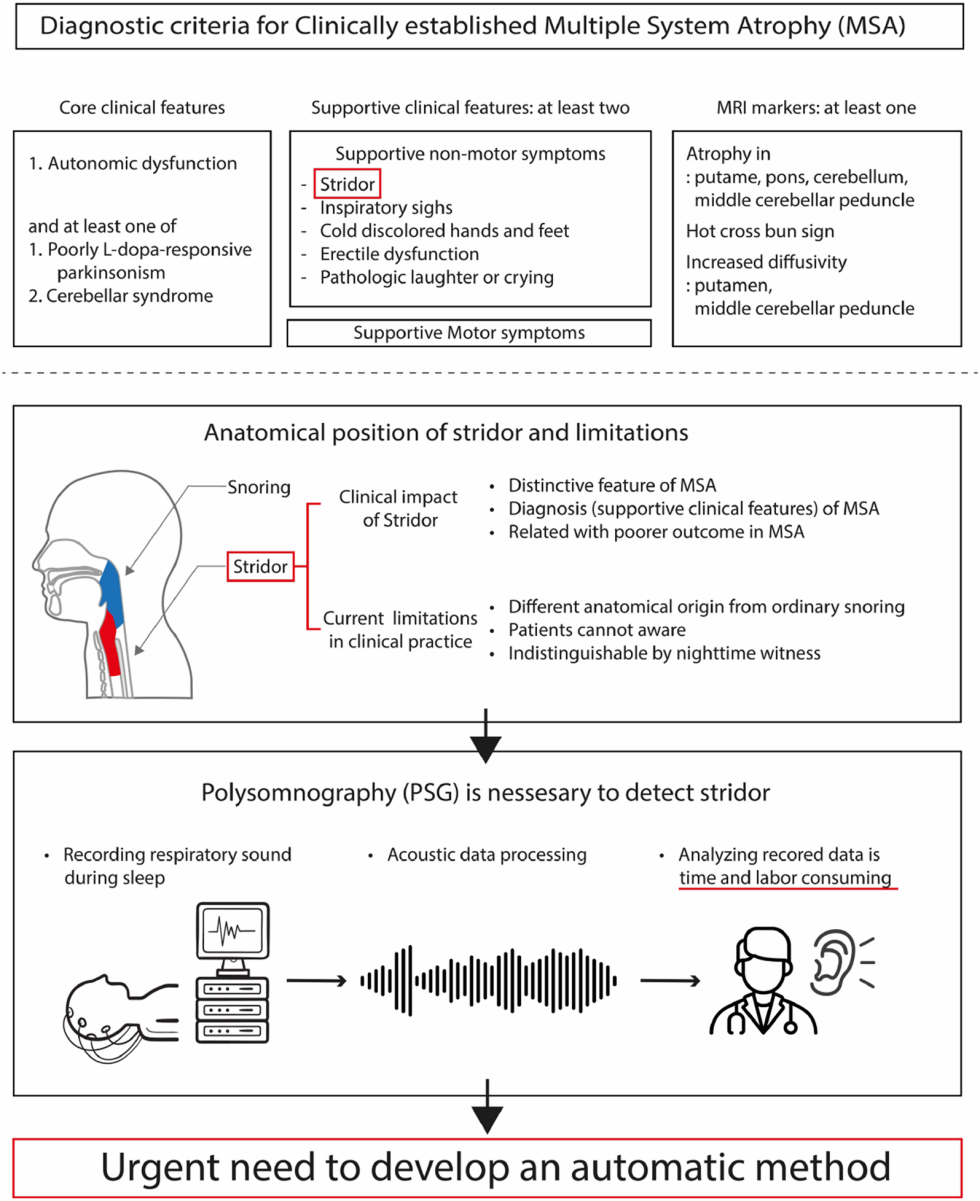
Current limitations of detecting and using stridor in clinical practice.

**Figure 2:**
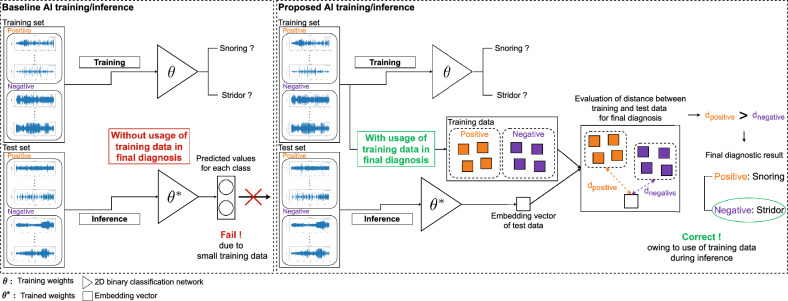
Overview of proposed PFL-SD and existing AI audio-based diagnosis methods. In the conventional method (baseline), $\theta ^{*}$ is correctly learned from the training data, but the network does not directly use training data for (post-training) diagnosis. In the proposed method, our network improves the performance by using training data even during diagnosis (post-training), that is, the distance between the training and inference samples is determined during classification. Hence, the proposed method improves its diagnostic performance with few training samples and correctly identifies stridor patches in an audio recording. The red box denotes the baseline method does not directly use training data for (post-training) diagnosis. The green box denotes the proposed method utilizes training data in the inference process (the training data is not evaluated additionally).

**Figure 3:**
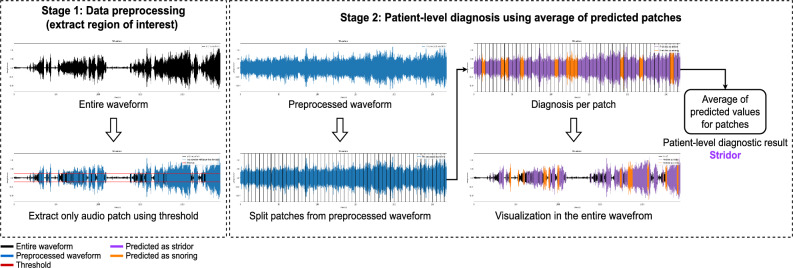
Diagram of proposed PFL-SD. The region of interest (ROI) is first extracted from sleep test audio signals of an individual. Then, SR is applied to the ROI to produce multiple audio patches for few-shot learning to diagnose stridor. By synthesizing diagnosis results for all the patches, a diagnosis is inferred. The diagnosis results for each patch allows visualization to interpret the results by identifying the patches suspected of stridor or snoring.

Multiple system atrophy (MSA) is a neurodegenerative disease characterized by progressive parkinsonism, ataxia, and autonomic dysfunction, including orthostatic hypotension and urinary dysfunction^1^. MSA diagnosis is challenging even for expert neurologists because patients with MSA show a variety of motor and non-motor symptoms throughout the disease progression^2^. At least two supportive clinical features are needed for diagnosing clinically established MSA, in addition to core clinical features (Fig. 1)^1^. Stridor is a high-pitched sound generated during inspiratory breathing mostly while sleeping and can be a distinctive non-motor indicator of MSA^3^. Early onset of stridor is associated with worse prognosis and unfavorable survival predictors in MSA^4^. As patients are often unaware of stridor, nighttime monitoring is necessary to identify stridor, but distinguishing between ordinary snoring and stridor is difficult (Fig. 1). Video-polysomnography (VPSG) is the most reliable diagnostic tool available for detecting stridor^3^. Stridor has unique acoustic features with a fundamental acoustic frequency of 260–330 Hz, which comprises formants and harmonics, in contrast to snoring, which has an irregularly shaped audio signal with neither formants nor harmonics^5^. However, identifying features and analyzing audio data are labor intensive and time consuming. To date, deep learning has not been used for binary classification of snoring and stridor. Considering the rarity of MSA and importance of stridor, an automatic method to detect stridor related to MSA should be developed using the few available samples. A recent advancement in few-shot learning has enabled the development of AI models with a few training samples, but it has not been applied to audio processing in the medical field^6–9^. Few-shot learning allows to integrate newly available training data during inference, thereby improving the diagnostic performance compared with other AI methods (Fig. 2). This learning strategy is particularly suited for developing classification applications with scarce training data, such as stridor data. The main contributions of this study are summarized as follows:

- We introduce a method to automatically diagnose stridor.
- We combine audio splitting and reintegration (SR) with few-shot learning in our method called patch-wise few-shot learning for sound detection (PFL-SD). This is the first method to incorporate these techniques into medical diagnosis based on audio signals, and we demonstrate the method validity.
- Compared with existing AI methods, PFL-SD improves the diagnostic performance for stridor even with scarce training data (achieving a stridor detection accuracy above 95%) while identifying and localizing suspected stridor patches in the audio recordings of a patient (Fig. 3). Hence, physicians may better interpret the method results with low inspection effort.

## Materials and methods

### Ethical approval

All the methods and experiments were designed and performed in accordance with the Declaration of Helsinki and relevant guidelines and regulations provided by the policies of Nature Portfolio journals. This study was approved by the Institutional Review Board of Samsung Medical Center (approval number: SMC 2022-02-030). Written informed consent from the patients was waived by the Institutional Review Board (Samsung Medical Center, Seoul, Republic of Korea) because we used anonymized retrospective data.

### Participants

Audio recordings from 65 participants were included in this study. Among the participants, 19 MSA patients had stridor and 46 patients had snoring without stridor. The demographics and clinical characteristics of the participants are listed in Table 1. The age, sex, unified Parkinson’s disease rating scale III, and Hoehn and Yahr scale were comparable between groups, and the disease duration was longer in patients with parkinsonism but without stridor.

### Data collection and ground-truth labeling

Data from eligible participants who underwent VPSG examinations were retrospectively selected for this study. We included participants who were diagnosed with MSA, idiopathic Parkinson’s disease, progressive supranuclear palsy, obstructive sleep apnea, and idiopathic rapid eye movement sleep behavior disorder at the time of VPSG. Sounds generated during sleep were collected from the participants using VPSG between October 2013 and May 2022. Overnight VPSG was performed using the Embla N7000 system (Medcare Flaga, Iceland). Behaviors and sounds from the subjects throughout the night were recorded using an infrared video camera and neck microphone placed below the chin. All the audio samples were digitized at 44,100 Hz with a 16-bit quantization level in stereo, and one sleep audio file was collected per subject. Stridor and snoring were distinguished by three experienced movement disorder and sleep medicine specialists (JH Ahn, J Youn, and EY Joo) and double-checked by blinded evaluation without clinical information. The patients were classified into with and without stridor groups. During the analysis, two cases elicited differing opinions among the experts. Following a thorough discussion, a consensus was achieved. A Kappa statistic of 0.949 (p < 0.001) underscored the high consistency among the experts’ evaluations. Patients with respiratory diseases or laryngeal abnormalities were excluded. Clinical and demographic data, including age, sex, disease duration, unified Parkinson’s disease rating scale III, and Hoehn and Yahr scale, were collected.

The normality of the data was evaluated using the Shapiro–Wilk test. Clinical and demographic features were represented by the mean and standard deviation. Differences among groups were determined using the analysis of variance, Student’s t-test, or chi-squared test. The results were considered significant for $p<$ 0.05. The statistical analyses were performed using the IBM SPSS software (version 28.0; IBM, USA) for the Microsoft Windows operating system.

**Table 1:** Demographics and clinical characteristics of participants.

	With stridor (*n* = 19)	Without stridor (*n* = 46)		
	MSA (*n* = 19)	Parkinsonism	Sleep disorders	P-value
		(IPD = 19, MSA = 6, PSP = 3)	(OSA = 10, iRBD = 8)	
Age (years)	62.8 ± 7.8	66.2 ± 7.4	58.3 ± 11.9	0.323
Sex (male, %)	57.9%	64.3%	72.2%	0.660
Disease duration (months)	28.4 ± 31.1	55.4 ± 46.7	−	0.041
UPDRS III	26.0 ± 15.6	23.1 ± 10.0	−	0.517
Hoehn and Yahr scale	2.5 ± 0.8	2.4 ± 0.6	−	0.447

PSP, progressive supranuclear palsy, IPD, idiopathic Parkinson’s disease; OSA, obstructive sleep apnea; iRBD, idiopathic rapid eye movement sleep behavior disorders; UPDRS III, unified Parkinson’s disease rating scale III.

**Table 2:** Data splitting for network training and test.

No. total training and test	No. training samples (*K*)					
(Patches are split at the patient-level)	**4**		**6**		**8**	
	Snoring	Stridor	Snoring	Stridor	Snoring	Stridor
No. patients of training (*K*)	4	4	6	6	8	8
No. patches of training (*K* $\times$ *P*)	120	120	180	180	240	240
No. patients of test (except for *K*)	42	15	40	13	38	11
No. patches of test (except for *K* $\times$ *P*)	1260	450	1200	390	1140	330

$$^{*}P$$ = 30 patches.

### Data preprocessing

We performed preprocessing of the audio recordings to extract regions of interest (i.e., regions in which stridor or snoring was pronounced) by using binary thresholds based on the audio volume. Subsequently, a new waveform was obtained by conserving only the waveform values above the threshold. Audio preprocessing is illustrated in the first stage of Fig. 3. We calculated the sound level (in decibels) of the entire waveform and set the threshold to 30% of this value. Preprocessing removed the patient’s silence and environmental noise, which accounted for more than one-third of the original audio recording.

### Data splitting for network training and testing

We considered 65 patients training data pairs $\mathscr {D}_{tr}:= \{(x_i,y_i)\}_{i=1}^{2K}$, where each class consisted of *K* pair sets (i.e., $\mathscr {D}_{tr}= \{(x_{i,0},y_{i,0})\}_{i=1}^{K} (=\mathscr {D}^0_{tr}) + \{(x_{i,1},y_{i,1})\}_{i=1}^{K} (=\mathscr {D}^1_{tr})$, where $\mathscr {D}^0_{tr}$ and $\mathscr {D}^1_{tr}$ denote the training sets for class 0 (snoring) and 1 (stridor), respectively), $x_i$ is the audio file of patient *i* after preprocessing, and $y_i$ denotes the class label (if $y_i=1$, patient *i* has suspected stridor; if $y_i=0$, patient *i* belongs to the normal group with snoring but without stenosis symptoms). The network was trained using the corresponding data, and the performance of the trained network was evaluated using the remaining data. *K* training data points for each class were randomly selected ($K=4,6,8$ in this study), and details of data splitting are listed in Table 2. For training and evaluation, Monte Carlo cross-validation^10^ was applied to obtain the mean and standard deviation over *M* trials ($M=10$ in this study).

### Network training and testing

**Figure 4:**
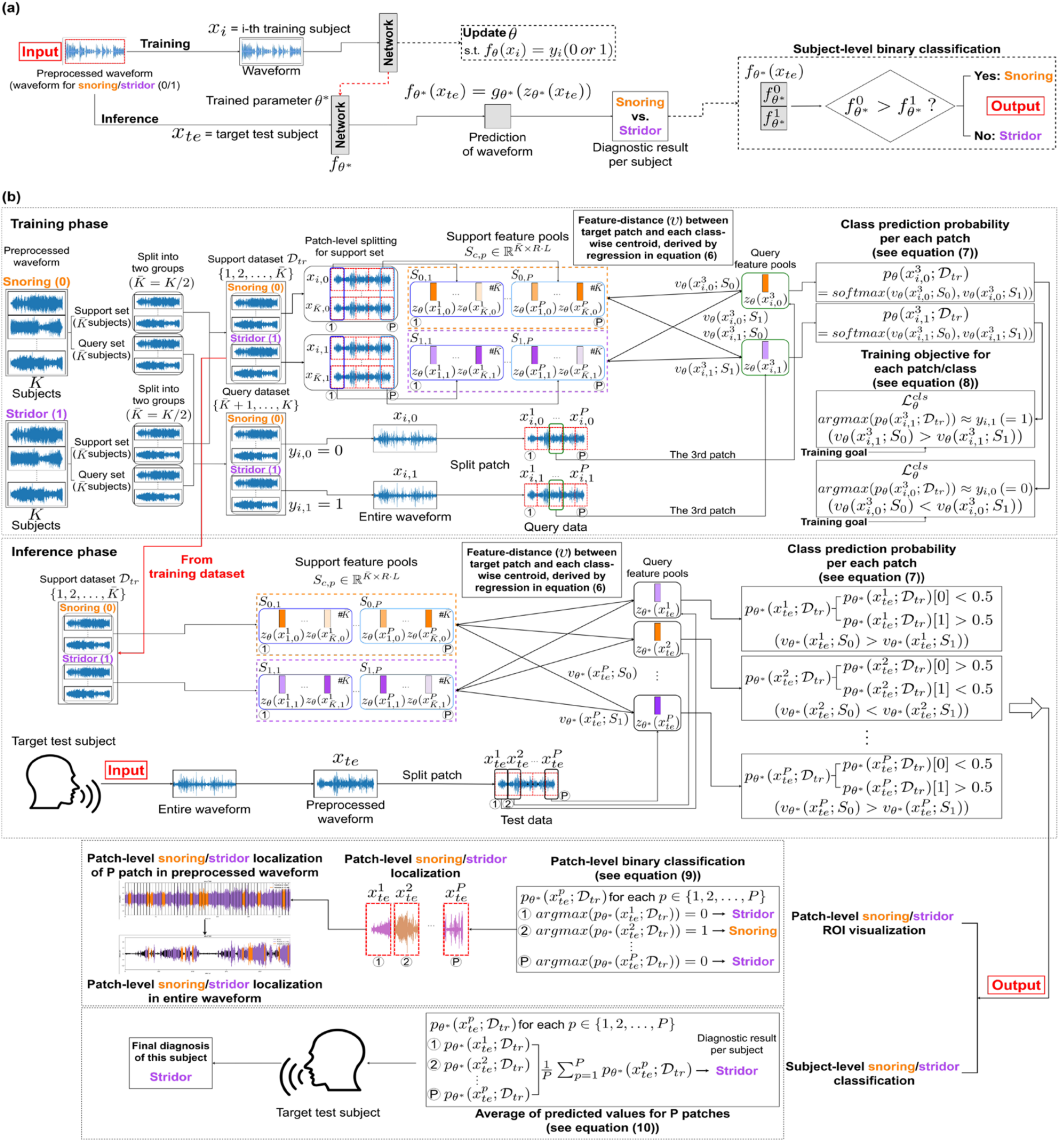
Diagram of proposed PFL-SD compared with conventional method. (**a**) The existing AI method (baseline) receives the patient’s entire audio recording, $x_{te}$, as the network input and provides the diagnosis result as probability vector $f_{\theta ^*}(x_{te})$. (**b**) The proposed PFL-SD splits the audio recording, $x_{te}$, into *P* patches $\{x^i_{te}\}_{i=1}^P$, provides a diagnosis probability estimate ($\{p_{\theta ^*}(x^i_{te})\}_{i=1}^P$) based on few-shot learning per patch, and performs patient-level diagnosis by merging the diagnosis results (Eq. (10)). The diagnosis of individual patches is performed through distance (Eq. (6)) comparison between the target sample and prototype representation of the training data support set per class^11^. As training data can be integrated as additional information even during inference, high diagnostic performance can be achieved even with few training samples available.

Owing to the nature of medical data, datasets are often insufficient for training AI models. To overcome this limitation, we devised a few-shot-learning-based patch segmentation^12–14^ for audio classification (stridor detection in this study). A comparison of the proposed method and the baseline is illustrated in Fig. 4. To introduce the proposed method, we describe the baseline and our proposal below.

### Baseline AI method

A general AI method for audio classification receives the preprocessed complete audio signal, $x_i$, as the input and learns to perform binary classification to provide ground-truth label $y_i \in \{0,1\}$ (training phase). After training, the network receives a test preprocessed complete audio signal, $x_{te}$, as the input and provides the binary classification prediction, $\hat{y} \in \{0,1\}$ (inference). Training and inference are outlined in Fig. 4a. These approaches were considered as the baseline (CNN14^15^) in this study.

**Training.** Network $f_{\theta }$ takes input $x_i$ and provides two-dimensional (2D) probability vector $f_{\theta }(x_i) \in \mathbb {R}^2$ as its output. Then, the baseline training is aimed to minimize the following loss function (i.e., determine network parameter $\theta ^*$ to minimize the loss):

1 $$\begin{aligned} \theta ^* = \underset{\theta }{\arg \min }\ \Big [ \sum _{i=1}^{2K} \mathscr {L}^{cls}_{\theta }(f_{\theta }(x_i),y_i) \Big ] \end{aligned}$$

where $\mathscr {L}^{cls}$ is a conventional classification loss (binary cross-entropy loss in this study). From training, the network output probability vector, $f_{\theta }(x_i) \in \mathbb {R}^2$, is learned to become a one-hot vector, where the value of the index of the inferred label, $y_i \in \{0,1\}$, is 1.

**Inference.** For inference after training, the index of the largest value of the network output probability vector, $f_{\theta ^*}(x_{te}) \in \mathbb {R}^2$, from the test audio signal, $x_{te}$, is considered as predicted class $\hat{y}_{\theta ^*}(x_{te})\in \{0,1\}$.

2 $$\begin{aligned} \hat{y}_{\theta ^*}(x_{te}) := \underset{}{\arg \max } \big ( f_{\theta }(x_{te})[0], f_{\theta }(x_{te})[1] \big ) \in \{0,1\} \end{aligned}$$

If the predicted class, $\hat{y}_{\theta ^*}(x_{te})$, is correctly estimated, it equals the ground-truth class label, $y_{te} \in \{0,1\}$ (i.e., $\hat{y}_{\theta ^*}(x_{te})=y_{te}$).

### Proposed AI method: PFL-SD

The proposed method is intended to achieve high diagnostic performance, even with few training samples, by applying patch-wise few-shot learning. Before describing training and inference, we explain the patch-wise audio splitting procedure. Preprocessed waveform audio signal *x* is divided into *P* sequential patches (i.e., splitting process in SR):

3 $$\begin{aligned} x \rightarrow (x^{1},x^{2},...,x^{P}) \end{aligned}$$

Because preprocessed audio signal *x* has a length of at least 150 s, we split an audio signal from 0 s to 150 s into $P=30$ patches of 5 s. Hence, we can detect stridor in each patch. In addition, when the diagnosis result is obtained by combining the patch results, the diagnostic performance is improved by using the patch-wise audio splitting owing to the increase in data diversity.

We perform training that uses each patch as a network input and generates the diagnosis results based on few-shot learning. For the set of *K* training samples per class, half of the set (i.e., $\{1,2,..., \bar{K}\}$ with $\bar{K}:=K/2$) is defined as a support set, and the other half (i.e., $\{\bar{K}+1,..., {K}\}$) is defined as the query set. The network is trained to minimize the average distance between the feature maps of the samples in the query and support sets. Specifically, the feature maps of the support set for class *c* can be expressed as matrix $S_{c}$, which is the collection of feature maps of dimension ${R \cdot L}$ for *P* patches and $\bar{K}$ objects in the support set of class *c* with dimension $\bar{K}P \times {R \cdot L}$:

4 $$\begin{aligned} S_{c}&:= \Big (S_{c,1}^{\top },S_{c,2}^{\top }, ..., S_{c,P}^{\top }\Big )^{\top } \in \mathbb {R}^{\bar{K}P \times {R \cdot L}} \end{aligned}$$

where

5 $$\begin{aligned} S_{c,p}&:= \Big (z_{\theta }(x^p_{1,c})^{\top },z_{\theta }(x^p_{2,c})^{\top },...,z_{\theta }(x^p_{\bar{K},c})^{\top }\Big )^{\top } \in \mathbb {R}^{\bar{K} \times {R \cdot L}}. \end{aligned}$$

Matrix $S_{c}$ in Eq. (4) is a set of feature maps generated by taking individual patches of the target group belonging to the support set with class *c* as inputs, and $S_{c,p}$ in Eq. (5) is the subset of $S_{c}$ such that only the feature maps of patch *p* are included. In Eq. (3), $z_{\theta }(x) \in \mathbb {R}^{R\cdot L}$ is the feature map of target network $f_{\theta }(x)=g_{\theta }(z_{\theta }(x))$, with *R* being the spatial resolution (i.e., height times weight) and *L* being the number of channels at an arbitrarily configurable layer in the network. We consider the feature map before the fully connected layers in the target network. In addition, $g_{\theta }$ is the set of the remaining layers, and $\theta$ is the network parameter for learning.

Given patch *p*, $x^p$, extracted from sample *x* in the query set, the distance in the feature domain between patch $x^p$ and support set $S_{c}$ for class *c* can be calculated as solution $v_{\theta }(x^p;S_{c})$ of ridge regression on the feature map as follows^16^:

6 $$\begin{aligned} v_{\theta }(x^p;S_{c})&:= \gamma \cdot \left\| z_{\theta }(x^p)(I-S_{c}^{\top }(S_{c}S_{c}^{\top }+\lambda I)^{-1})S_{c} \right\| ^2 \end{aligned}$$

where $\gamma$ and $\lambda$ are constants that control the scale and rank of the distance, respectively. We set the constants according to the original setup^6^.

A smaller value (distance) increases the probability that the target query patch, $x^p$, belongs to class *c*, as depicted in Fig. 4. Accordingly, whether target query patch $x^p$ belongs to either class, 0 (snoring) or 1 (stridor), can be expressed as a probability through distance comparison with the support set for each class as follows:

7 $$\begin{aligned} p_{\theta }(x^p;\mathscr {D}_{tr})&:= \Big ( \frac{\exp (-v_{\theta }(x^p;S_{0}))}{\sum _{c' \in \{0,1\}} \exp (-v_{\theta }(x^p;S_{c'}))}, \frac{\exp (-v_{\theta }(x^p;S_{1}))}{\sum _{c' \in \{0,1\}} \exp (-v_{\theta }(x^p;S_{c'}))} \Big ) \end{aligned}$$

**Training.** The detailed training of the proposed PFL-SD is shown in Fig. 4b. By applying a conventional classification loss to the class prediction probability vector, $p_{\theta }(x^p;\mathscr {D}_{tr})$, based on the distance between samples, the network is trained to obtain a one-hot vector that refers to ground-truth class $y_{i,c}$ (i.e., *c*) for each input query patch $x^p_{i,c}$:

8 $$\begin{aligned} \theta ^* = \underset{\theta }{\arg \min }\ \Big [ \sum _{p=1}^{P} \sum _{c=0}^{1}\sum _{i=\bar{K}+1}^{K} \mathscr {L}^{cls}_{\theta }\big (p_{\theta }(x^p_{i,c};\mathscr {D}_{tr}),y_{i,c}\big ) \Big ] \end{aligned}$$

**Inference.** From the index information corresponding to the maximum value, the learned probability vector, $p_{\theta ^*}(x^p;\mathscr {D}_{tr})$, provides a prediction of the class to which input patch $x^p$ belongs. Given the *P* patches per patient, we integrate the *P* diagnosis results (i.e., reintegration in SR) to obtain a patient-level diagnosis. As a straightforward approach, the prediction is derived as $\hat{y}_{\theta ^*}(x_{te};\mathscr {D}_{tr}) \in \{0,1\}$ in Eq. (9) from average probability vector $\bar{p}_{\theta ^*}(x_{te};\mathscr {D}_{tr})$ of *P* class prediction probability vectors acquired from the *P* patches (i.e., $\{x^i_{te}\}_{i=1}^P$) for preprocessed test audio signal $x_{te}$.

9 $$\begin{aligned} \hat{y}_{\theta ^*}(x_{te};\mathscr {D}_{tr}) := \underset{}{\arg \max } \big ( \bar{p}_{\theta ^*}(x_{te};\mathscr {D}_{tr})[0], \bar{p}_{\theta ^*}(x_{te};\mathscr {D}_{tr})[1] \big ) \in \{0,1\} \end{aligned}$$

where

10 $$\begin{aligned} \bar{p}_{\theta ^*}(x_{te};\mathscr {D}_{tr}) := \frac{1}{P}\sum _{p=1}^{P}p_{\theta ^*}(x^p_{te};\mathscr {D}_{tr}). \end{aligned}$$

Figure 4b illustrates the final diagnosis and abnormal patch visualization of PFL-SD.

**Comparison between proposed method and baseline.** The proposed method has two main differences with the existing baseline.

- When designing the class probability prediction vector, the baseline ($f_{\theta }(x_{te})$ in Eq. (2)) only considers whether target sample $x_{te}$ belongs to the corresponding class but not the correlation with other samples. Unlike the baseline, the proposed method can achieve a higher diagnostic performance because it considers information from other samples in addition to $x_{te}$. Hence, different from $f_{\theta }(x_{te})$ in Eq. (2) of the baseline, class probability prediction vector $\bar{p}_{\theta ^*}(x_{te};\mathscr {D}_{tr})$ in Eq. (9) of the proposed method uses training sample $\mathscr {D}_{tr}$ (i.e., support set $S_c$ of class *c* in $\mathscr {D}_{tr}$) during inference. Externally using training samples for inference prevents overfitting caused a small training set in the conventional method (i.e., Eqs. (1) and (2)). This is because the conventional method uses training samples exclusively to obtain the training parameters. In this study, we applied few-shot learning to stridor detection and demonstrated its effectiveness.
- We improved the performance of few-shot learning by redesigning training and inference to be applied to each of the *P* audio patches to then integrate the inference results into a patient-level diagnosis (Eq. (10)). In addition to the clinical implications of accurate stridor detection, the proposed patch-wise few-shot learning is an innovative approach.

### Experimental settings and implementation details

For the audio recordings of sounds (length between 724 s and 20,638 s) generated by each patient during sleep, we performed preprocessing as illustrated in stage 1 of Fig. 3. For each preprocessed audio signal, a segment of 150 s was extracted. This segment length is commonly used for the input in various classification networks. The proposed method split this segment into *P* patches (i.e., patches were divided by patient-level), received individual patches as inputs, and aggregated the *P* results for final diagnosis. We set *P* to 30, and thus each patch had a length of $150/30=5$ s.

For the input, we converted every signal into a Log-Mel spectrogram and used the resulting 2D representation as input. The Log-Mel spectrogram has commonly been used as the input instead of the sound signal^15,17–19^, and we adopted this strategy. For existing AI methods, the Log-Mel spectrogram 2D representation of each 150 s signal was obtained and used as input. For the proposed AI method, *P* Log-Mel spectrogram 2D representations of the 5 s patches were obtained from each 150 s signal and used as inputs.

The baseline was trained as follows. We used CNN14^15^ as the backbone. This network consisted of six convolution blocks, with each block comprising two convolutional layers with a kernel size of $2\times 2$. After the last block, global average pooling^20^ was applied to extract the 2D feature map of each channel. We used the same implementation reported by Song et al.^21^. For training, we set the minibatch size to 8, the number of epochs to 500, the binary cross-entropy loss with an initial learning rate of 0.01, and an adaptive moment estimation^22^ for optimization. We also applied transfer learning^23^ to set the initial network parameters to those pre-trained on the AudioSet dataset^24^. We set the audio sampling rate to 22,050 (Hz), window size to 2048, hop size to 512, and window type to Hann^15^.

The proposed method was trained as follows. We used ResNet12^6–9^ as the backbone. It consisted of four residual blocks, with each residual block comprising three convolutional layers with a kernel size of $3\times 3$, and a $2\times 2$ max-pooling layer was applied after the first three blocks. We used the same implementation for ResNet12 reported by Wertheimer et al.^6^. The proposed PFL-SD was trained with a minibatch size of 8, number of epochs of 500, binary cross-entropy loss with an initial learning rate of 0.01, and stochastic gradient descent^25^ for optimization. We also applied transfer learning to use the pre-trained parameters from the mini-ImageNet dataset^26^ for initialization. Similar to the baseline setup, we set the audio sampling rate to 22,050 (Hz), window size to 2048, hop size to 512, and window type to Hann^15^.

## Results

### Evaluation measures of classification performance

To evaluate the stridor detection performance of the proposed PFL-SD (i.e., binary classification of snoring or stridor), we used the area under the curve (AUC) of the receiver operating characteristic (ROC) curve, accuracy, sensitivity, specificity, precision, and F1 score. In the ROC curve, we selected the decision threshold as 0.5, which is the most commonly used, and calculated the true positive (*TP*), true negative (*TN*), false positive (*FP*), and false negative (*FN*) rates based on that threshold. Then, the accuracy, sensitivity, specificity, precision, and F1 scores were calculated as follows:

$$\begin{aligned} Accuracy&= \frac{(TN+TP)}{(TN+TP+FN+FP)}, \\ Sensitivity&= \frac{(TP)}{(TP+FN)}, \\ Specificity&= \frac{(TN)}{(TN+FP)}, \\ Precision&= \frac{(TP)}{(TP+FP)}, \\ F1\, score&= 2 \, \frac{Precision \times Sensitivity}{Precision+Sensitivity}. \end{aligned}$$

**Table 3:** Diagnostic performance of existing and proposed methods in terms of accuracy. We evaluated the patient-level mean diagnostic performance for detection of stridor or snoring. The mean and standard deviation were obtained from 10-fold Monte Carlo validation.

Method	No. training samples		
	4	6	8
Existing method 1 (ResNet18)^27^	0.686 ± 0.09	0.753 ± 0.06	0.768 ± 0.08
Existing method 2 (ResNet50)^27^	0.609 ± 0.16	0.734 ± 0.11	0.751 ± 0.06
Existing method 3 (DenseNet201)^28^	0.679 ± 0.07	0.760 ± 0.05	0.792 ± 0.05
Existing method 4 (MobileNetV2)^29^	0.674 ± 0.11	0.715 ± 0.08	0.725 ± 0.11
Existing method 5 (VGG16)^30^	0.705 ± 0.11	0.723 ± 0.15	0.801 ± 0.11
Existing method 6 (YAMNet)^31^	0.676 ± 0.11	0.717 ± 0.10	0.786 ± 0.07
Existing method 7* (CNN14, baseline)^15^	0.786 ± 0.06	0.802 ± 0.05	0.869 ± 0.07
Proposed method (Ver. 1)	0.854 ± 0.08	0.902 ± 0.04	0.935 ± 0.05
Proposed method (Ver. 2; PFL-SD)	**0.874** ± **0.12**	**0.934** ± **0.05**	**0.961** ± **0.03**

The highest values in the results for each number of training samples are shown in bold.

$^*$ Baseline with the highest performance among existing methods considered in this study.

**Table 4:** Performance of each network according to number of training samples. The mean and standard deviation were obtained from 10-fold Monte Carlo validation.

No. training samples	Method	Accuracy	Sensitivity	Specificity	Precision	F1 score
4	Baseline^15^	0.786 ± 0.06	0.600 ± 0.24	0.852 ± 0.12	0.657 ± 0.18	0.576 ± 0.15
	Proposed (Ver. 1)	0.854 ± 0.08	0.800 ± 0.16	0.874 ± 0.11	0.739 ± 0.20	0.749 ± 0.12
	Proposed (Ver. 2)	**0.874** ± **0.12**	**0.813** ± **0.20**	**0.895** ± **0.15**	**0.799** ± **0.25**	**0.784** ± **0.17**
6	Baseline^15^	0.802 ± 0.05	0.746 ± 0.15	0.820 ± 0.09	0.595 ± 0.10	0.647 ± 0.08
	Proposed (Ver. 1)	0.902 ± 0.04	**0.877** ± **0.08**	0.910 ± 0.04	0.767 ± 0.08	0.815 ± 0.06
	Proposed (Ver. 2)	**0.934** ± **0.05**	0.862 ± 0.16	**0.958** ± **0.04**	**0.875** ± **0.10**	**0.862** ± **0.11**
8	Baseline^15^	0.869 ± 0.07	0.782 ± 0.15	0.895 ± 0.09	0.730 ± 0.19	0.734 ± 0.14
	Proposed (Ver. 1)	0.935 ± 0.05	0.909 ± 0.06	0.942 ± 0.08	0.857 ± 0.15	0.871 ± 0.07
	Proposed (Ver. 2)	**0.961** ± **0.03**	**0.936** ± **0.08**	**0.968** ± **0.03**	**0.906** ± **0.10**	**0.916** ± **0.06**

The highest values in the results for each number of training samples are shown in bold.

### Performance of existing and proposed diagnosis methods

The proposed PFL-SD comprises audio SR and diagnosis based on few-shot learning. In addition, the method has versions 1 and 2 for few-shot learning without and with SR, respectively. The diagnostic performance of the proposed and existing methods was compared. Note that existing methods lack SR and few-short learning. We evaluated seven methods with different backbones: ResNet18^27^, ResNet50^27^, DenseNet201^28^, MobileNetV2^29^, VGG16^30^, YAMNet^31^, and CNN14^15^. 10-fold Monte Carlo validation was applied in network training and testing to evaluate the diagnostic performance across 10 trials by randomly selecting a sample from the dataset for training and the remaining samples to construct an evaluation dataset per fold. Four, six, and eight training samples were considered per class. The existing and proposed methods used the same Log-Mel spectrogram as input for a fair comparison.

The experimental results from the performance evaluation are listed in Table 3. We confirmed that the best-performing existing method was the one based on CNN14, which was thus designated as the baseline. Version 1 of the proposed method outperformed the baseline, while version 2 outperformed version 1. These results consistently appeared for different numbers of training samples. Therefore, the proposed method (version 2) achieved superior stridor diagnostic performance compared with existing methods. In addition, the validity of combining SR and few-shot learning was verified because version 2 outperformed version 1 that lacked SR.

**Figure 5:**
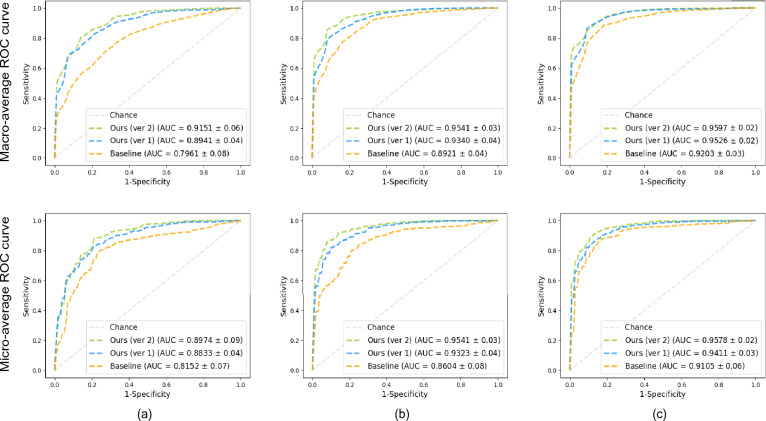
Receiver operating characteristic (ROC) curve of each network for (a) four, (b) six, and (c) eight training samples.

To further compare the performance of the proposed and existing methods, we investigated various performance indicators in addition to accuracy. The results are presented in Fig. 5 (AUC), Table 5 (AUC with 95% confidence interval (CI)), and Table 4 (other measures). Table 4 shows the accuracy, sensitivity, and specificity of the methods. In most cases, the proposed technique showed a higher sensitivity and specificity than the baseline. In addition, the performance improvement was confirmed by the F1 score, verifying the superiority of the proposed method, which did not reflect class imbalance. Figure 5 and Table 5 show the receiver operating characteristic curves and AUC (with 95% CI) of the proposed method and baseline, respectively. The proposed method consistently improved the performance for all the decision thresholds compared with the baseline, indicating its lack of bias regardless of the threshold. Table 6 shows the confusion matrices from which the results for Tables 3 and 4 were derived. The matrices supported the reliability of the results.

**Table 5:** Diagnostic performance of baseline and proposed methods in terms of AUC. We evaluated the patient-level mean diagnostic performance for detection of stridor or snoring. The mean, standard deviation, and 95% confidence interval were obtained from 10-fold Monte Carlo validation.

Metric	Method	No. training samples		
		4	6	8
Macro-avg AUC (95% CI)	Baseline	0.7961 ± 0.08 (0.747, 0.846)	0.8921 ± 0.04 (0.867, 0.917)	0.9203 ± 0.03 (0.902, 0.939)
	Proposed (Ver. 1)	0.8941 ± 0.04 (0.869, 0.919)	0.9340 ± 0.04 (0.909, 0.959)	0.9526 ± 0.02 (0.940, 0.965)
	Proposed (Ver. 2)	**0.9151 ± 0.06 (0.878, 0.952)**	**0.9541 ± 0.03 (0.935, 0.973)**	**0.9597 ± 0.02 (0.947, 0.972)**
Micro-avg AUC (95% CI)	Baseline	0.8152 ± 0.07 (0.772, 0.859)	0.8604 ± 0.08 (0.811, 0.910)	0.9105 ± 0.06 (0.873, 0.948)
	Proposed (Ver. 1)	0.8833 ± 0.04 (0.858, 0.908)	0.9323 ± 0.04 (0.908, 0.957)	0.9411 ± 0.03 (0.923, 0.960)
	Proposed (Ver. 2)	**0.8974 ± 0.09 (0.842, 0.953)**	**0.9541 ± 0.03 (0.935, 0.973)**	**0.9578 ± 0.02 (0.945, 0.970)**

The highest values in the results for each number of training samples are shown in bold.

**Table 6:** Confusion matrix of each network according to number of training samples. The mean and standard deviation were obtained from 10-fold Monte Carlo validation.

Method	No. training samples											
	4				6				8			
Baseline^15^	Confusion matrix		Predicted		Confusion matrix		Predicted		Confusion matrix		Predicted	
			Snoring	Stridor			Snoring	Stridor			Snoring	Stridor
	Actual	Snoring	35.8 ± 5.07	6.2 ± 5.07	Actual	Snoring	32.8 ± 3.61	7.2 ± 3.61	Actual	Snoring	34 ± 3.3	4 ± 3.3
		Stridor	6 ± 3.68	9 ± 3.68		Stridor	3.3 ± 2.11	9.7 ± 2.11		Stridor	2.4 ± 1.65	8.6 ± 1.65
Proposed (Ver. 1)	Confusion matrix		Predicted		Confusion matrix		Predicted		Confusion matrix		Predicted	
			Snoring	Stridor			Snoring	Stridor			Snoring	Stridor
	Actual	Snoring	36.7 ± 4.32	5.3 ± 4.32	Actual	Snoring	36.4 ± 1.65	3.6 ± 1.65	Actual	Snoring	35.8 ± 3.01	2.2 ± 3.01
		Stridor	3 ± 2.31	12 ± 2.31		Stridor	1.6 ± 1.07	11.4 ± 1.07		Stridor	1 ± 0.67	10 ± 0.67
Proposed (Ver. 2)	Confusion matrix		Predicted		Confusion matrix		Predicted		Confusion matrix		Predicted	
			Snoring	Stridor			Snoring	Stridor			Snoring	Stridor
	Actual	Snoring	37.6 ± 5.8	4.4 ± 5.8	Actual	Snoring	38.3 ± 1.7	1.7 ± 1.7	Actual	Snoring	36.8 ± 1.23	1.2 ± 1.23
		Stridor	2.8 ± 2.94	12.2 ± 2.94		Stridor	1.8 ± 1.93	11.2 ± 1.93		Stridor	0.7 ± 0.95	10.3 ± 0.95

### Visualization of diagnosis results using proposed method

**Figure 6:**
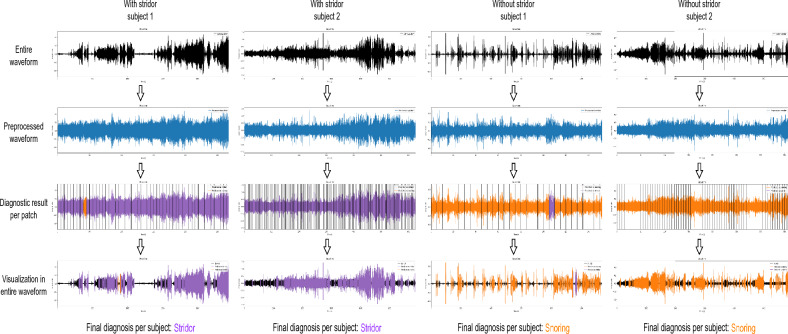
Visualization of real-time diagnosis results obtained from proposed PFL-SD. Applying patch segmentation to an audio source, the proposed PFL-SD method performs separate diagnosis on every patch. Merging the patch-level results provides the patient-level diagnosis, and the individual stridor diagnosis results per patch can be visualized. Hence, physicians can manually review only stridor audio patches to confirm the diagnosis of stridor. Additional visualization examples of snoring and stridor are shown in Supplementary Figure S1.

The proposed PFL-SD classifies an audio signal into a stridor or snoring case while allowing to visualize the patches in the input audio signal to evaluate the diagnosis result. Figure 6 illustrates the sequential introduction of the four main processes in the proposed PFL-SD, providing the visualization results and verifying the visualization capability. The proposed method extracts audio patches during preprocessing and performs separate diagnoses. These results are merged to provide patient-level diagnosis, and the individual diagnosis results can be displayed on the original audio source to visualize the patches containing classification information of stridor or snoring. This visualization strategy may allow physicians to focus on patches containing stridor information (purple patches in Fig. 6) for accurate diagnosis, likely shortening the time required for stridor confirmation.

We provide videos of a participant with or without stridor in Supplementary Files S3 and S4 of the supplementary information. The videos show the diagnosis results of the proposed PFL-SD along with the audio source. The sounds related to stridor (i.e., high-pitched inspiratory breathing sounds) appear only in sections with positive stridor classification, indicating the superiority of PFL-SD over other methods and its potential clinical applicability. Additional explanations and examples of Supplementary Files S3 and S4 are shown in Supplementary Figure S2.

## Discussion

We first developed binary classification using an AI method for detecting stridor, which is an important non-motor symptom in MSA. Considering the rarity of MSA and stridor, developing an AI method with high diagnostic performance is challenging, but we obtained high performance by applying few-shot learning. Even with few training samples, the proposed method achieved a detection accuracy above 95%. In addition to stridor detection, the proposed method could locate the audio patches showing stridor in real time, thus providing physicians with additional assistance for interpreting and employing the diagnosis results.

MSA is a rare neurogenerative disease with a prevalence of 3.4–4.9 cases per 100,000 persons^3^. The prevalence of stridor in MSA varies from 12% to 42% depending on the disease stage^32^. Stridor is an important non-motor symptom of MSA that facilitates both diagnosis and prognosis. Stridor is a supportive non-motor symptom of clinically established MSA and a distinctive feature of MSA mimicking^1^. In addition, early presence of stridor is an independent predictor of shorter survival^4^. Continuous positive airway pressure or tracheostomy is recommended for managing stridor, with the latter possibly improving patient survival^3^. Therefore, the timely detection and management of stridor are crucial for patients with MSA, but there is no gold standard for stridor diagnosis. Moreover, nighttime monitoring is required because most patients show stridor during sleep, and they may be unaware of its occurrence. Although nighttime monitoring allows the detection of abnormal breathing, stridor detection should be confirmed on VPSG because stridor is often confused with ordinary snoring, which is also common in MSA. Consequently, stridor detection is labor intensive and time consuming for a physician, who should manually review acoustic data recorded from VPSG. Therefore, an automatic method to detect stridor and differentiate it from ordinary snoring should be developed. The proposed automatic AI method for stridor detection can achieve a high accuracy of 96.1%.

The proposed method can facilitate stridor detection if embedded into a smartphone or voice recorder. Meira et al.^33^ reported the recording of breathing sounds using a smartphone to support diagnosis. They found that recording breathing sounds may help physicians by enabling early stridor detection and MSA diagnosis. In addition, 5.2% of MSA patients have exhibited stridor^4^, which can be used for screening or detecting prodromal symptoms of MSA. Although a very low positive rate is expected considering the low prevalence of MSA and stridor, automatic stridor detection using our method may have a low cost and negligible labor burden. To date, no method for automatic stridor diagnosis has been developed, rendering the physician’s examination indispensable. Thus, the patient with MSA should visit a hospital for suspected stridor, and VPSG is conducted. The physician then manually reviews the audio recordings and determines the presence of stridor. Consequently, this approach is only applied when the patient visits the hospital after MSA diagnosis and when stridor is suspected. This hinders detection when an individual is not diagnosed with MSA or has no suspicion of stridor, likely missing timely diagnosis and proper early treatment. We have shown the feasibility of developing a method for automatic and accurate stridor diagnosis. Hence, stridor can be diagnosed even outside hospital settings, and an individual can visit the hospital and undergo a detailed examination if stridor is diagnosed. As a result, deterioration caused by this neurodegenerative disease may be mitigated at an early stage.

Medical audio classification using AI and few training samples has previously been demonstrated^21,34–38^. However, our method is the first one to use few training samples for accurate *classification of snoring and stridor* using AI. We achieve high diagnostic performance by applying *few-shot learning* to binary classification using audio recordings in the medical field. Existing AI solutions^21,34–38^ have performed audio classification without considering correlations between samples during inference, like in the baseline scheme illustrated in Fig. 2, which simply outputs a probability vector for the classes of an input sample while neglecting the similarity between samples. Conventional classification without sample correlation tends to perform poorly when few training samples are available^39^. Given scarce training data, overfitting on the decision boundary of the classifier can occur. Externalizing the decision boundary rather than learning it internally through few-shot learning allows inferring the class of a sample by calculating the distance between its location and the class location obtained from the training samples. Hence, the learning efficiency and inference performance can be enhanced with only few training samples. The performance improvement is due to the information on the input test sample (conventional method) being used along with information from all the training samples for classification. Accordingly, we apply few-shot learning for the first time to stridor detection to support MSA diagnosis (Fig. 2) and demonstrate improved diagnostic performance over other AI methods using few training samples.

Although our proposed method has shown promising results, since this is a prototype to perform snoring and stridor binary classification with small data using few-shot learning, there are limitations to be addressed in future work. First, the number of stridor patients used in our study was unsatisfactory. We used 65 patients (19 with stridor and 46 without stridor) as the dataset of our study, and we tried to collect snoring and stridor patients at similar rates, but only 29% of all patients (i.e., 65 patients) were stridor patients due to the rarity of MSA and stridor^3^. Therefore, the proportion of snoring patients (71%) was higher than the proportion of stridor patients (29%) (i.e., the ratio of snoring to stridor is about 7 to 3), resulting in slightly higher specificity (although we were able to derive high sensitivity by arbitrarily controlling the threshold, we set it to the default threshold of 0.5.). However, since the proportion of snoring patients in the actual diagnosis (i.e., breath sound test through polysomnography) of MSA patients will be more than about 70%^40^, we expect that models with high specificity will increase the diagnostic success rate of MSA patients (i.e., with snoring and stridor, which are common sleep breathing problems). Second, since the purpose of our study was to develop a tool to distinguish between snoring and stridor, we could not evaluate various audio recording data (i.e., excluding snoring and stridor data). Future studies should investigate the applicability of our proposed method to problems such as stridor and other abnormal breathing sounds (e.g., wheeze, crackle, etc.) classification by collecting various audio recording data. And if a large number of data is collected, it is necessary to additionally consider a method suitable for classifying a large dataset (e.g., contrastive learning^41–44^).

Although our study has some limitations, we apply audio SR to few-shot learning for the first time. SR splits a sleep test audio recording into multiple patches and classifies them. The classification across patches is then merged to obtain patient-level diagnosis, as illustrated in Fig. 3. Accordingly, through SR, we obtain multiple diagnosis results from a patient and achieve superior performance owing to the audio patch diversity. In addition, SR improves diagnostic performance and helps physicians interpret the AI results by providing diagnosis results for patches with suspected stridor in the audio source. The patches containing stridor information can be visualized, as reported in the Results section.

## Conclusion

We implemented automatic stridor detection using few-shot learning and patch splitting for audio processing in the medical field using an AI method. The proposed PFL-SD showed high-performance stridor detection, an important MSA indicator, even with fewer training samples than those required for conventional AI methods. The proposed PFL-SD merged diagnosis results from multiple patches extracted from an audio signal. The obtained patient-level result improved the diagnostic performance, and the patch-level results enabled the visualization of stridor-suspected patches for confirmation by a physician. A patient shows short stridor periods in an audio recording from a sleep test despite the diagnosis being positive. Until now, a physician had to analyze the entire audio recording to discover periods with suspected stridor, resulting in a costly and burdensome evaluation. The proposed method may allow physicians to confirm stridor by simply listening to the patches with positive stridor diagnosis, considerably accelerating the diagnosis confirmation. Although the proposed PFL-SD was only validated for stridor diagnosis on sleep test data, we expect to extend the method to various sound applications in the medical field, especially those with challenging data collection, to demonstrate its superiority and clinical utility in future work.

## Supplementary Information

Please download Supplementary Files S3 and S4 and play in the video player (legend for Supplementary Files S3 and S4 are shown in Supplementary Figure S5). Supplementary Files S3 and S4 were created by Ju Hwan Lee using the OpenCV (version 3.4.2) library^45^ in Python (version 3.7.9).

## Supplementary Information

Supplementary Information 1.

Supplementary Information 2.

Supplementary Information 3.

Supplementary Information 4.

Supplementary Information 5.

## Data Availability

The main data supporting the results of this study are reported within the paper. The raw datasets from Samsung Medical Center are protected to preserve patient privacy, but they can be made available upon reasonable request if approval is obtained from the corresponding Institutional Review Board. For the request, please contact Jin Whan Cho at jinwhan.cho@samsung.com.

## Code availability

The code used in this study is available at https://github.com/kskim-phd/PFL-SD.

## References

[1] Wenning GK (2022). The movement disorder society criteria for the diagnosis of multiple system atrophy. Mov. Disorders.

[2] Marsili L, Giannini G, Cortelli P, Colosimo C (2021). Early recognition and diagnosis of multiple system atrophy: Best practice and emerging concepts. Exp. Rev. Neurotherap..

[3] Cortelli P (2019). Stridor in multiple system atrophy: Consensus statement on diagnosis, prognosis, and treatment. Neurology.

[4] Giannini G (2016). Early stridor onset and stridor treatment predict survival in 136 patients with msa. Neurology.

[5] Koo DL, Lee JY, Joo EY, Hong SB, Nam H (2016). Acoustic characteristics of stridor in multiple system atrophy. PloS one.

[6] Wertheimer, D., Tang, L. & Hariharan, B. Few-shot classification with feature map reconstruction networks. In *Proceedings of the IEEE/CVF Conference on Computer Vision and Pattern Recognition*, 8012–8021 (2021).

[7] Ye, H.-J., Hu, H., Zhan, D.-C. & Sha, F. Few-shot learning via embedding adaptation with set-to-set functions. In *Proceedings of the IEEE/CVF Conference on Computer Vision and Pattern Recognition*, 8808–8817 (2020).

[8] Tian, Y., Wang, Y., Krishnan, D., Tenenbaum, J. B. & Isola, P. Rethinking few-shot image classification: a good embedding is all you need? In *European Conference on Computer Vision*, 266–282 (Springer, 2020).

[9] Lee, K., Maji, S., Ravichandran, A. & Soatto, S. Meta-learning with differentiable convex optimization. In *Proceedings of the IEEE/CVF conference on computer vision and pattern recognition*, 10657–10665 (2019).

[10] Xu Q-S, Liang Y-Z (2001). Monte carlo cross validation. Chemom. Intell. Lab. Syst..

[11] Snell J, Swersky K, Zemel R (2017). Prototypical networks for few-shot learning. Adv. Neural Inf. Process. Syst..

[12] Sinha R, Tranter SE, Gales MJ, Woodland PC (2005). The cambridge university March 2005 speaker diarisation system. Interspeech.

[13] Meignier S, Moraru D, Fredouille C, Bonastre J-F, Besacier L (2006). Step-by-step and integrated approaches in broadcast news speaker diarization. Comput. Speech Language.

[14] Tranter SE, Reynolds DA (2006). An overview of automatic speaker diarization systems. IEEE Trans. Audio Speech Language Process..

[15] Kong Q (2020). Panns: Large-scale pretrained audio neural networks for audio pattern recognition. IEEE/ACM Trans. Audio Speech Language Process..

[16] Hoerl AE, Kennard RW (1970). Ridge regression: Biased estimation for nonorthogonal problems. Technometrics.

[17] McFee, B. *et al.* librosa: Audio and music signal analysis in python. In *Proceedings of the 14th python in science conference*, vol. 8, 18–25 (Citeseer, 2015).

[18] Choi, K., Fazekas, G. & Sandler, M. Automatic tagging using deep convolutional neural networks. arXiv preprint arXiv:1606.00298 (2016).

[19] Kong Q (2019). Weakly labelled audioset tagging with attention neural networks. IEEE/ACM Trans. Audio Speech Language Process..

[20] Lin, M., Chen, Q. & Yan, S. Network in network. arXiv preprint arXiv:1312.4400 (2013).

[21] Song J (2022). Detection and differentiation of ataxic and hypokinetic dysarthria in cerebellar ataxia and parkinsonian disorders via wave splitting and integrating neural networks. PloS one.

[22] Kingma, D. P. & Ba, J. Adam: A method for stochastic optimization. arXiv preprint arXiv:1412.6980 (2014).

[23] Pan SJ, Yang Q (2009). A survey on transfer learning. IEEE Trans. Knowl. Data Eng..

[24] Gemmeke, J. F. *et al.* Audio set: An ontology and human-labeled dataset for audio events. In *2017 IEEE International Conference on Acoustics, Speech and Signal Processing (ICASSP)*, 776–780 (IEEE, 2017).

[25] Ruder, S. An overview of gradient descent optimization algorithms. arXiv preprint arXiv:1609.04747 (2016).

[26] Ren, M. *et al.* Meta-learning for semi-supervised few-shot classification. arXiv preprint arXiv:1803.00676 (2018).

[27] He, K., Zhang, X., Ren, S. & Sun, J. Deep residual learning for image recognition. In *Proceedings of the IEEE Conference on Computer Vision and Recognition* 45, 770–778 (2016).

[28] Huang, G., Liu, Z., Van Der Maaten, L. & Weinberger, K. Q. Densely connected convolutional networks. In *Proceedings of the IEEE Conference on Computer Vision and Pattern Recognition*, 700–4708 (2017).

[29] Sandler, M., Howard, A., Zhu, M., Zhmoginov, A. & Chen, L.-C. Mobilenetv2: Inverted residuals and linear bottlenecks. In *Proceedings of the IEEE Conference on Computer Vision and Pattern Recognition*, 4510–4520 (2018).

[30] Simonyan, K. & Zisserman, A. Very deep convolutional networks for large-scale image recognition. arXiv preprint arXiv:1409.1556 (2014).

[31] Plakal, M. & Ellis, D. Yamnet. https://github.com/tensorflow/models/tree/master/research/audioset/yamnet (2020).

[32] Fanciulli A, Wenning GK (2015). Multiple-system atrophy. New England J. Med..

[33] Meira B, Barbosa R, Mendonça M (2020). Can you hear your patient sleep? smartphones and modern technologies in the detection of nocturnal stridor and msa diagnosis. Mov. Disord. Clin. Pract..

[34] Xie J (2021). Audio-based snore detection using deep neural networks. Comput. Methods Progr. Biomed..

[35] Swarnkar, V. R., Abeyratne, U. R. & Sharan, R. V. Automatic picking of snore events from overnight breath sound recordings. In *2017 39th Annual International Conference of the IEEE Engineering in Medicine and Biology Society (EMBC)*, 2822–2825 (IEEE, 2017). 10.1109/EMBC.2017.8037444 29060485

[36] Sun, J. *et al.* Snorenet: Detecting snore events from raw sound recordings. In *2019 41st Annual International Conference of the IEEE Engineering in Medicine and Biology Society (EMBC)*, 4977–4981 (IEEE, 2019). 10.1109/EMBC.2019.8857884 31946977

[37] Jiang Y, Peng J, Zhang X (2020). Automatic snoring sounds detection from sleep sounds based on deep learning. Phys. Eng. Sci. Med..

[38] Shen F (2020). Detection of snore from osahs patients based on deep learning. J. Healthc. Eng..

[39] Power, A., Burda, Y., Edwards, H., Babuschkin, I. & Misra, V. Grokking: Generalization beyond overfitting on small algorithmic datasets. arXiv preprint arXiv:2201.02177 (2022).

[40] Iranzo A (2007). Sleep and breathing in multiple system atrophy. Curr. Treat. Options Neurol..

[41] Li, M. *et al.* Contrastive unsupervised learning for speech emotion recognition. In *ICASSP 2021-2021 IEEE International Conference on Acoustics, Speech and Signal Processing (ICASSP)*, 6329–6333 (IEEE, 2021).

[42] Fonseca, E., Ortego, D., McGuinness, K., O’Connor, N. E. & Serra, X. Unsupervised contrastive learning of sound event representations. In *ICASSP 2021-2021 IEEE International Conference on Acoustics, Speech and Signal Processing (ICASSP)*, 371–375 (IEEE, 2021).

[43] Saeed, A., Grangier, D. & Zeghidour, N. Contrastive learning of general-purpose audio representations. In *ICASSP 2021-2021 IEEE International Conference on Acoustics, Speech and Signal Processing (ICASSP)*, 3875–3879 (IEEE, 2021).

[44] Soni PN, Shi S, Sriram PR, Ng AY, Rajpurkar P (2022). Contrastive learning of heart and lung sounds for label-efficient diagnosis. Patterns.

[45] Bradski G, Kaehler A (2008). Learning OpenCV: Computer vision with the OpenCV Library.

