# Supplementary material for: Automatic stridor detection using small training set via patch-wise few-shot learning for diagnosis of multiple system atrophy
Source: Sci Rep. 2023 Jul 5;13:10899. doi: 10.1038/s41598-023-37620-0 (PMC10323004; doi:10.1038/s41598-023-37620-0)

Supplementary Figure S1. Examples of visualization results of PFL-SD for (a) snoring and (b) stridor subjects.

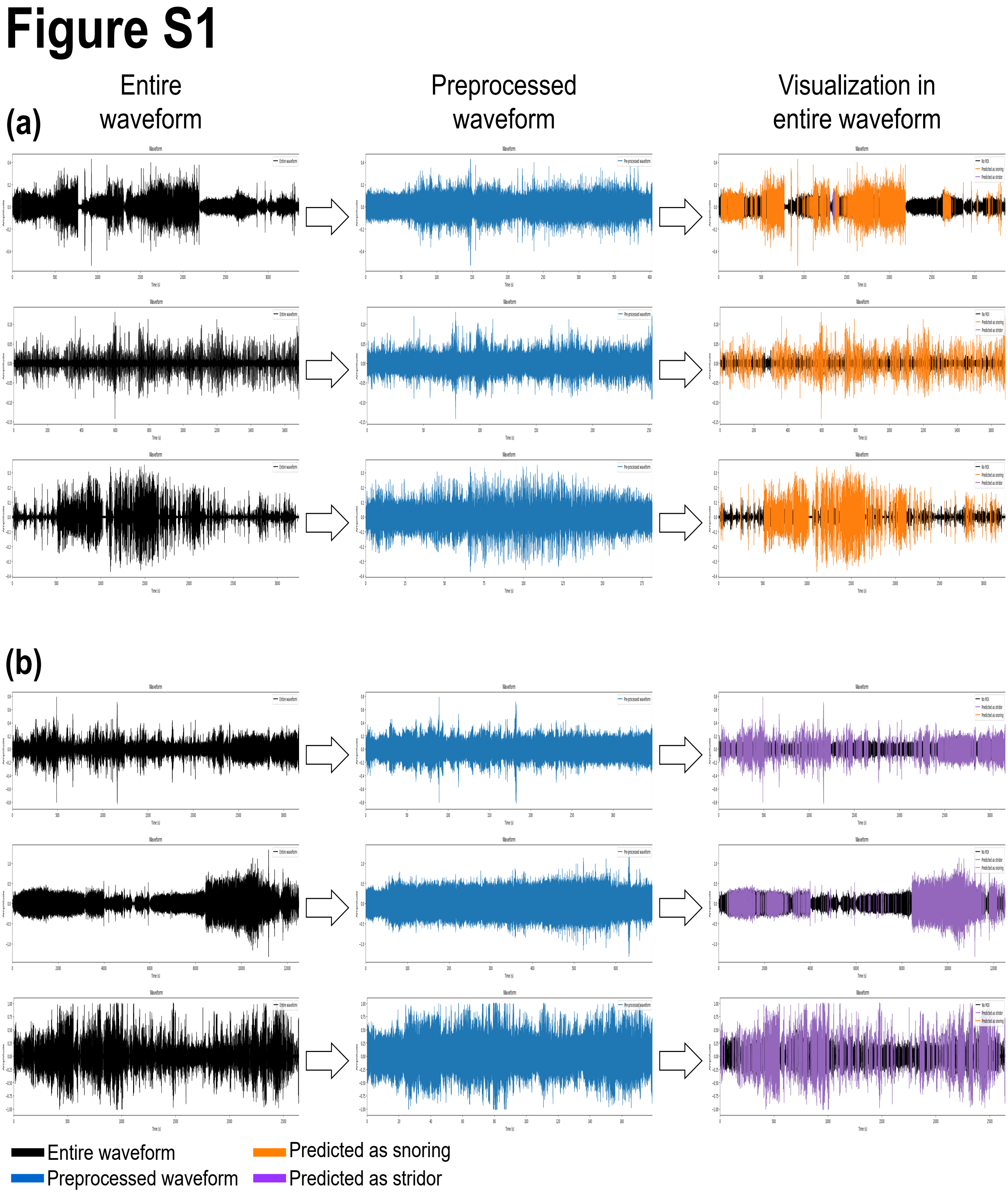

Supplement: Supplementary file 1 — Supplementary Information 1. [file 41598_2023_37620_MOESM1_ESM.pdf]
