# Supplementary material for: Automatic stridor detection using small training set via patch-wise few-shot learning for diagnosis of multiple system atrophy
Source: Sci Rep. 2023 Jul 5;13:10899. doi: 10.1038/s41598-023-37620-0 (PMC10323004; doi:10.1038/s41598-023-37620-0)

Supplementary Figure S2. Examples of video results of PFL-SD for (a) snoring (Supplementary File S3) and (b) stridor (Supplementary File S4) subjects. When the video is played with the video player, the bar moves every second and shows the diagnosis results of the proposed PFL-SD along with the audio source. Detailed implementation configuration and code can be found at `https://github.com/kskim-phd/PFL-SD`.

Figure S2

(a) Supplementary File S3 (44,100 Hz, 18:57)

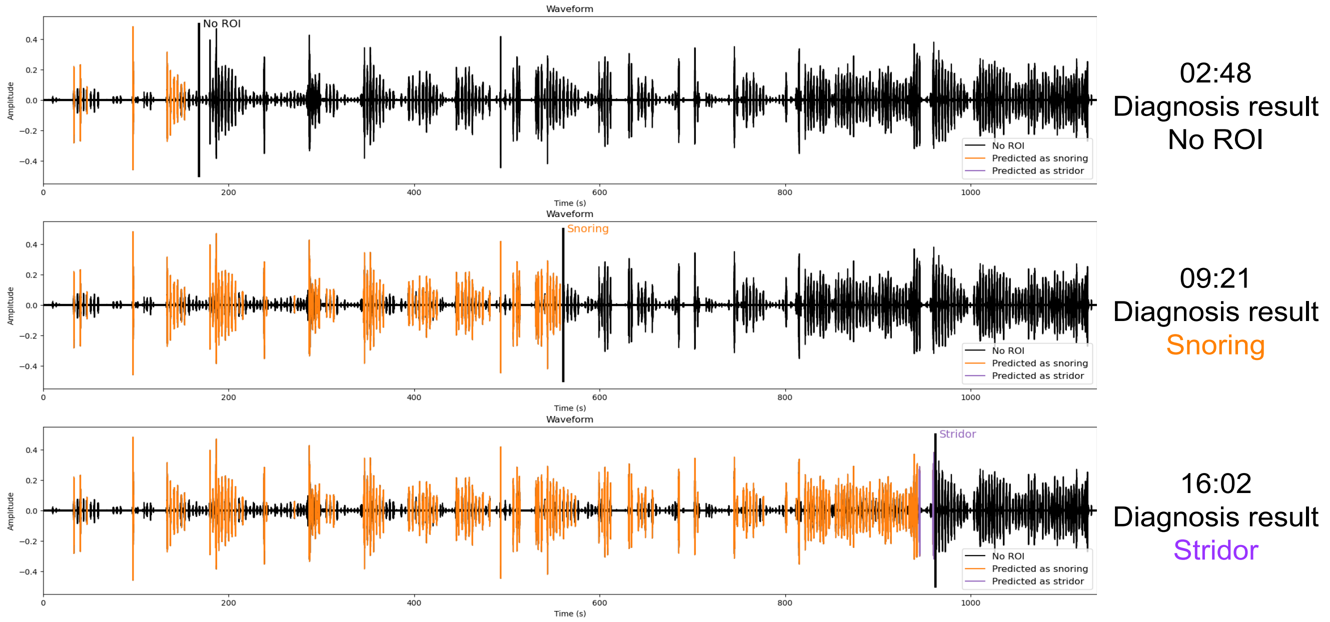

(b) Supplementary File S4 (44,100 Hz, 40:52)

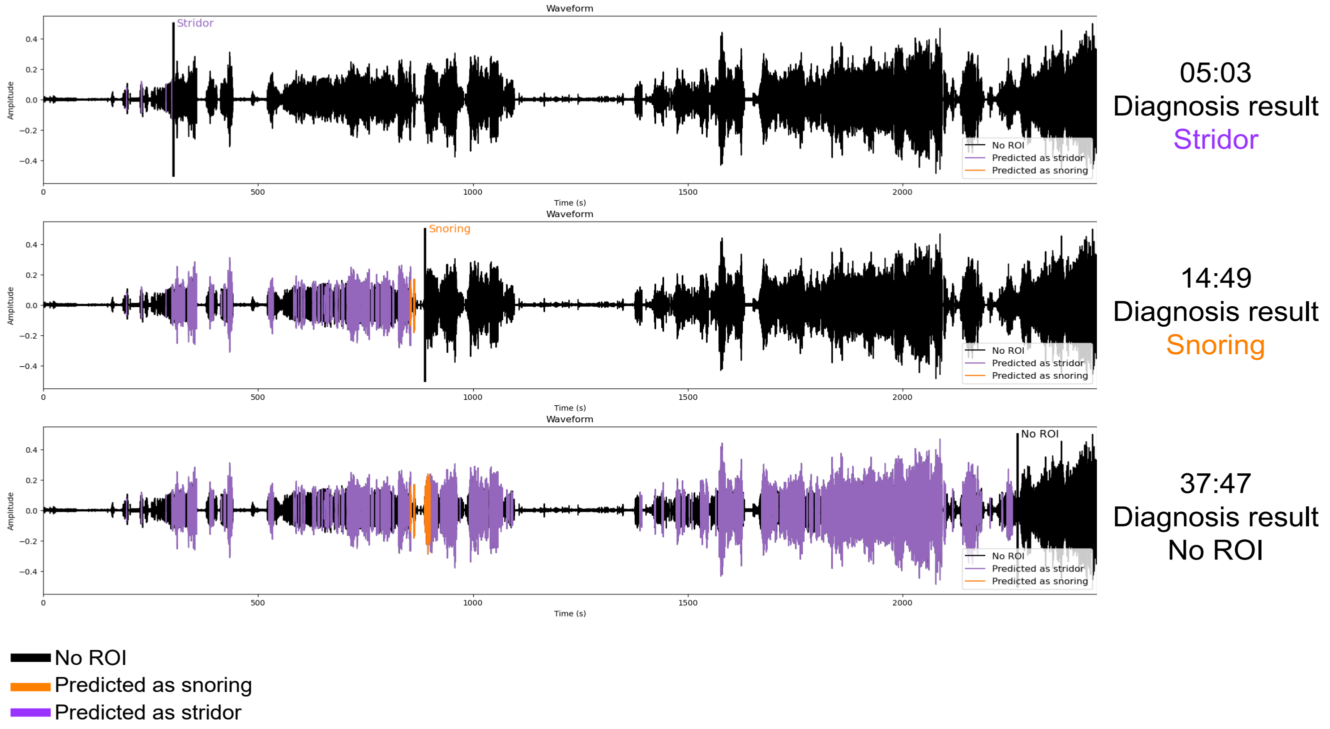

Supplement: Supplementary file 2 — Supplementary Information 2. [file 41598_2023_37620_MOESM2_ESM.pdf]
