# Supplementary figures and images for: Automatic stridor detection using small training set via patch-wise few-shot learning for diagnosis of multiple system atrophy
Source: Sci Rep. 2023 Jul 5;13:10899. doi: 10.1038/s41598-023-37620-0 (PMC10323004; doi:10.1038/s41598-023-37620-0)

# Figure S5

- 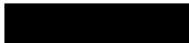 No ROI
- 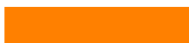 Predicted as snoring
- 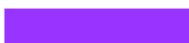 Predicted as stridor

Supplement: Supplementary file 5 — Supplementary Information 5. [file 41598_2023_37620_MOESM5_ESM.pdf]
